# Pharmacoeconomic studies in Nepal: the need of the hour

**DOI:** 10.3389/fphar.2014.00272

**Published:** 2014-12-08

**Authors:** Mahip Acharya

**Affiliations:** Independent ResearcherKathmandu, Nepal

**Keywords:** pharmacoeconomics, reimbursement, health-care decision making, me-too products, cost-effectiveness

Pharmacoeconomics is a rapidly expanding field with interests from the whole gamut of health-related personnel, spanning from hospital administrators to policy makers (Drummond et al., 2003). It incorporates various analytical techniques that deal with costs and outcomes of pharmaceutical interventions employed in prevention, diagnosis, treatment and management of diseases. Pharmacoeconomic evaluation is concerned with the comparison of two or more drug therapies with respect to their costs and consequences. Considered a branch of health economics, pharmacoeconomics limits itself to the pharmaceutical facet of health-care system. Cost-minimization analysis, cost-effectiveness analysis, cost-utility analysis, and cost-consequence analysis are chief types of pharmacoeconomic studies (Drummond, 2006). These studies are carried out from the perspectives of patient, payer, or society, these perspectives differing in the type of costs included in the studies (Drummond, 2006). That pharmacoeconomic studies are highly regarded by health-care experts and policy makers for basing health policy decisions, emphasize their significance (Greenberg et al., 1999). These studies are of immense significance to pharmaceutical industries as well, the research and development of newer drugs and critical decisions regarding them being influenced by results of pharmacoeconomic studies in many instances (DiMasi et al., 2001).

Pharmacoeconomic studies are a regular in most of the countries, both developed and developing. They are carried out in academic institutions—with vested academic interest—as well as by certain organizations (Drummond et al., 2003). In countries like Australia and Canada, the results of these studies for new pharmaceuticals need to be submitted to the Government—these countries have mandatory pharmacoeconomic guidelines—for reimbursement purpose (Hjelmgren et al., 2001). In US, mandatory guidelines on Government' part are not available; however, insurance companies have their separate set of guidelines for reimbursement on their part—health insurance coverage in US comes from many private insurance companies (third party payers) (Hjelmgren et al., 2001). Asian countries such as Japan, Korea, China, Singapore, Hongkong, and Taiwan are also making a rapid progress in pharmacoeconomic aspect of health system—they are still behind the Western countries, though (Doherty et al., 2004). India is also realizing the gravity of pharmacoeconomic studies in the realm of health sector, and requisite steps are being taken for consolidating pharmacoeconomic studies in the country (Thakkar and Billa, 2013). International Society for Pharmacoeconomics and Outcomes Research (ISPOR), with membership from 115 countries, has been contributing to the spread of significance of pharmacoeconomic studies within the health-care system. Pharmacoeconomic studies are thus starting to gain a foothold in the global health sector, with many countries starting to incorporate these studies in their health-care system.

Nepal is a small, mountainous country situated in the southeastern region of Asia. It is sandwiched between India and China. Nepal is a least developed country which is ranked 157th in Human Development Index (UNDP, 2011), this lower ranking reflecting the socio-economic status of the country. The protracted political imbroglio and the time-and-again insurgency by political parties and combatant groups—Maoists' insurgency being the latest and arguably the most threatening—have hampered the progress of the country. Weak governance, incapacitated public sector, slow recovery from civil conflicts, inadequate infrastructures—chiefly, transportation and electricity—and mediocre industrial performance have been the seminal factors for poor performance of Nepal in socio-economic index (Asian Development Bank, 2009). There is a considerable disparity in socioeconomic status among people from different topographical regions (Asian Development Bank, 2009). Marred by political turmoil and sluggish socio-economic progress, Nepal has not done much to make its mark on socio-economic and political front in the international arena. Health sector has also faced the repercussions of these socio-economic and political upheavals.

Health sector of Nepal is toddling its way and has had its share of achievements and disappointments in recent years. Millennium Development Goals (MDGs) for maternal mortality rate and child mortality rate have already been achieved by Nepal (Government of Nepal, 2013). However, there are other dimensions of health sector that are not fully addressed, not least is the inequitable distribution of health services among different geographical and socio-economic groups. There is a considerable disparity in the health services available for people of rural and urban areas, for people from privileged families, and those from marginalized ones, and for people from different age and gender groups (Government of Nepal, 2013). Moreover, the quality of health services is not up to the international standards. Also, a lion's share of public sector health expenditure comes from foreign donors (Ministry of Health and Population, 2010), thereby accentuating the fragile state of the health sector. Pharmaceutical division, among various sections of the health sector, has not done anything monumental, but is headed in the right direction (Budhathoki, 2012). Increasing number of pharmaceutical industries, production of high quality medicines, and their lower price compared to imported medicines are a few positives of Nepal's pharmaceutical sector; however, it is also strewn with shortcomings such as mediocre research and development, absence of raw materials (active pharmaceutical ingredients) manufacturers, and fierce competition with foreign-manufactured products (Budhathoki, 2012). Against this backdrop of pharmaceutical sector, pharmacoeconomic studies are virtually non-existent in Nepal.

Government's expenditure in health is a meager 5.3 percent of GDP and per capita health expenditure is USD18.09, as per 2006 data (Ministry of Health and Population, 2010). If this is juxtaposed against the data from developed countries like the United States, Japan—per capita health care expenditure being USD8,608 for US and USD3,958 for Japan (Davidson, 2013)—the state of public spending in health of Nepal becomes all the more apparent. In addition, more than 55 percent of total health expenditure comes from out-of-pocket health expenditure (Ministry of Health and Population, 2010). With such limited spending in health sector on public sector's part and with people dishing out money for health services as per their economic status, pharmacoeconomic studies have become highly desirable to ascertain accessibility of one and all to health services.

National Drug Policy-1995 of Nepal has no mention of pharmacoeconomic studies (Department of Drug Administration, 1995). These studies have been viewed as a prerogative of health sector of developed countries, with Nepal still grappling to provide basic health facilities and essential medicines to the public. On the contrary, the significance of these studies becomes more glaring in such situations, where wise spending in health services, including medicines, is paramount. The likes of cost-effectiveness and cost-minimization analysis help determine cost effective medicines for different diseases. The findings from these studies could be internalized, which could lead to availability of better medicines with considerable saving of public sector's fund. Moreover, the country is gearing up for materializing community-based health insurance program, with the aim of establishing universal health coverage; in fact, pilot programs have already been carried out in a few districts (GIZ, 2012). Formulary making and reimbursement decisions are next steps to follow, and pharmacoeconomic evaluation is a contributing factor in this regard. This indeed calls for a sea change in the perception toward pharmacoeconomic studies and their legitimacy in Nepal. That there is not a single regional chapter, affiliated with ISPOR, in Nepal underscores the state of pharmacoeconomic studies in Nepal. It is high time health experts and policy makers in the country had a proper look at pharmacoeconomics and its inherent benefits.

Unlike other countries, pharmacoeconomic studies in Nepal have not been a dear of academia and industrial sector. Although academic institutions dealing with pharmacy education are on the rise in the country, they have still not been inclined toward pharmacoeconomic studies. The chief reason for this is lack of funding for such studies, which should come from the pharmaceutical industries or public sector. In a scenario where pharmaceutical industries of Nepal are competing with foreign industries for merely making an impact in Nepalese pharmaceutical market, delving into pharmacoeconomic studies, and providing funding for these studies remain an uncharted territory for them. The research and development division of pharma industries in Nepal is in its incipient stage, so newer drug molecules or newer combinations being discovered in Nepal is not a recurring headline in media. In fact, clinical trials in Nepal are few and far between, with serious issues with methodology, ethics and the rationale behind them (Sathian, 2011). In such a situation, piggy-back pharmacoeconomic evaluations, the type that is carried out alongside clinical trials (O'Sullivan et al., 2005), are out of equation, for standardization of clinical trials is needed first in present context of Nepal. In the same vein, enough justice cannot be done at present to naturalistic or effective pharmacoeconomic trials, where data are collected in a real-world situation and which is a good substitute for the one done together with clinical trials (Revicki and Frank, 1999; Garrison et al., 2007). Research and development of new pharmaceutical products is still in its early days in Nepal; as a result, the pharmacoeconomic studies pertaining to new products are not what the country is seeking for at the moment.

Pharmacoeconomic studies of the existing medicines are more pertinent in present context of Nepal. Pharmaceutical market of Nepal is rife with me-too products (Budhathoki, 2012), which provide minimal extra benefit than the respective prototype product. Most of these products need to be sieved off so that the budget and labor can be directed toward such products that carry an added benefit compared to its competing products. Pharmacoeconomic evaluation of these products can be exploited to ward off such products. Although the price of these products are less than the imported ones, this lower price emanates from market competition without any role of value of these products. Value-based pricing, however, is unlikely in the near future in Nepal, so the primary concern has to be ensuring cost-effectiveness. Observational studies (Dreyer et al., 2010) and economic modeling (Soto, 2002) are few such techniques that can be exploited at present for this very purpose. Observational studies may be prospective or retrospective in nature, both of which can be carried out in Nepal with few limitations. Health database available at hospitals and health centers can be utilized, which when combined with cost-of-illness and epidemiological data make for a full-fledged pharmacoeconomic evaluation. One such study—the prospective one—has already been performed for effectiveness analysis of pharmaceutical intervention in diabetes mellitus at a tertiary care center in eastern Nepal (Das et al., 2011). However, such studies are very few in number, and they have been conducted solely with academic interest and not with an eye to inform the health care decision making.

This brings us to the lack of pharmacoeconomic studies influencing policy level decision making and lack of funding for them in Nepal. That pharmaceutical industries of Nepal are currently not in a position to fund such studies further complicate the situation. Nevertheless, these studies are of urgent need in the country, and the initiatives need to be taken. This has to come from the public sector, which carries out different health programs in the country. Health sector of Nepal is assisted by fundings from different International Organizations for smooth running of most of its programs (Ministry of Health and Population, 2010). Moreover, the country has adopted a sector-wide approach in health sector, where funds are collected and directed toward health programs under the leadership of the Government. The public sector has to take charge in pharmacoeconomic studies as well, primarily by providing funding for these studies. It may not be feasible to fund large number of such studies; therefore, considerations also need to be given toward establishing a regional chapter in the country and adopting findings, with adjustments, of studies conducted in other countries, wherever possible.

Economic evaluation of pharmaceutical products is a must in developing countries like Nepal. Disseminating the findings of pharmacoeconomic studies and assimilating them in decision-making process can lead to manufacture of cost-effective medicine and better health budget allocation in the country.

## Conflict of interest statement

The author declares that the research was conducted in the absence of any commercial or financial relationships that could be construed as a potential conflict of interest.
